# Action and interaction strategies for enhancing upward referral of obstetric emergencies from community health centres in the eastern cape

**DOI:** 10.3389/fgwh.2026.1798049

**Published:** 2026-04-09

**Authors:** Final Zimkhitha Juqu, Zamadonda Xulu-Kasaba, Olivia Baoraopetse Baloyi

**Affiliations:** 1 Department of Public Health Medicine, College of Health Sciences, University of KwaZulu-Natal, Durban, South Africa; 2 Department of Nursing, Walter Sisulu University, Mthatha, South Africa

**Keywords:** community health center (CHC), eastern cape province, maternal mortality, obstetric emergencies, referral system

## Abstract

**Introduction:**

Effective upward referral of obstetric emergencies from community health centres to higher-level facilities is critical for reducing maternal and neonatal mortality. In resource-limited settings such as the Eastern Cape, South Africa, referral systems are frequently constrained by transport shortages, communication gaps and staffing limitations. Although referral policies exist, limited evidence describes the practical strategies frontline healthcare workers use to maintain functional referral processes under these conditions.

**Methods:**

This study analysed the action and interaction strategies used to facilitate effective upward referral of obstetric emergencies from CHCs. A qualitative approach, embedded within a broader grounded theory inquiry and guided by the Straussian method, was employed. Data were collected through focus group discussions, in-depth interviews, observations and document analysis.

**Results:**

Healthcare workers employed a range of adaptive strategies to overcome systemic referral barriers. These included improvised transportation and logistical arrangements, informal communication pathways, bedside clinical improvisation, strategic reallocation of human and material resources and deliberate adaptation of referral protocols. These practices were collaborative, context-responsive, and frequently extended beyond formal procedures to prioritise patient survival.

**Discussion:**

Despite structural constraints, frontline healthcare workers sustain obstetric referral systems through dynamic and context-responsive practices. These findings highlight the critical role of provider agency and adaptive decision-making in maintaining continuity of emergency obstetric care and strengthening maternal and neonatal outcomes in resource-limited health systems.

## 1 Introduction and background

Globally, maternal and neonatal mortality remain a pressing challenge with an estimated 260,000 women dying from pregnancy and childbirth complications in 2023 [World Health Organization (WHO) 2025]. The challenge disproportionately affects resource-constrained settings (1). Timely and effective emergency obstetric referrals play a critical role in improving survival outcomes (2), particularly for women with complications that cannot be managed at primary or community health levels (3). In these resource-limited settings, delays and inefficiencies in the referral system significantly contribute to preventable maternal and neonatal deaths (4).

**Table 1:** Profile of the participants.

Group	Name	Roles
CHC 1, FGD 1	Zikhona	Female Midwives
	Asenathi	
	Lindiwe	
CHC 2, FGD 2	Weliswa	Female Midwives
	Ncebakazi	
	Lihle	
	Nozibele	
CHC 3. FGD 3	Alice	2 Male Midwives, 1 Female
	Frank	
	Ndodiphela	
CHC 4, FGD 4	Clarissa	Female Midwives
	Zoe	
	Pat	
	Florence	
CHC 5, FGD 5	Emihle	Female Midwives
	Lelethu	
	Mandisa	
Hospital 1, FGD 6	Zoleka	Female Midwives
	Lunga	
	Natasha	
	Khazi	
	Elsie	
Hospital 2, FGD 7	Nancy	Female Midwives
	Joyce	
	Abonga	
Hospital 2, FGD 8	Bantu	1 Male Midwife, 3 females
	Nosiphiwo	
	Nqobile	
	Siyanda	
Hospital 3, FGD 9	Zama	1 Male Midwife, 2 Females
	Buhle	
	Talita	
Hospital 3, FGD 10	Nkosazana	Female Midwives
	Xolile	
	Sesethu	
	Bathandwa	
Hospital 1, FGD 11	Jamjam	2 Male Midwives, 2 Females
	Kholeka	
	Nomsa	
	Wazini	
Hospital 2; FGD 12	Mpumi	Female Midwives
	Xhasi	
	Nontle	
	Nomzamo	
Hospital 3; FGD 13	Diliza	3 Female Midwives, 1 Male
	Nokwanda	
	Thenjiwe	
	Zandisile	
Medical Officers	Andrew (IDI 1)	5 Male, 3 Females
	Sandiso (IDI 2)	
	Fefekazi (IDI 3)	
	Zakhele (IDI 4)	
	Busisiwe (IDI 5)	
	Langa (IDI 6)	
	Khanyisile (IDI 7)	
	Kwanele (IDI 8)	
EMS Paramedics	Mandy (IDI 9)	2 Female Paramedics, 1 Male
	Kwanda (IDI 10)	
	Ntombifuthi (IDI 11)	

This critical concern resonates strongly in South Africa, where upward referral of obstetric emergencies from community health centres (CHCs) to higher-level hospitals providing comprehensive emergency obstetric care is central to reducing mortality (5). Yet, referral processes, particularly in the Eastern Cape, are often fragmented, resulting in avoidable delays and poor outcomes (6, 7). While national guidelines and policies outline referral protocols (5, 8, 9), implementation is frequently inconsistent. In practice, midwives, paramedics, doctors and even families often rely on improvised solutions to navigate challenges (10). These everyday coping mechanisms, although not formally recognised, are critical for sustaining referral systems under difficult conditions.

From a grounded theory (GT) perspective, such practices can be understood as action/interaction strategies, which are purposeful processes through which individuals and groups collaborate to overcome systemic constraints (11). Yet, despite their importance, the strategies remain under-documented and poorly understood within the South African context in relation to obstetric emergency referral. The need for deeper insights is especially critical in the Eastern Cape, where rurality, long distances, ambulance shortages and staff constraints severely hinder timely referral (34).

## 2 Problem statement

Conditions that require immediate escalation, such as obstetric haemorrhage and hypertension, among others, are leading underlying causes of maternal mortality in South Africa (12) and in the Eastern Cape (13). Ensuring rapid recognition and referral of these conditions is therefore vital to reducing preventable maternal deaths. In the Eastern Cape, common modifiable mortality factors contributing to maternal deaths include delays or failures in referral, misjudgement of severity and inadequate referral documentation (13). Reflecting these challenges, the province recorded the highest Institutional Maternal Mortality Ratio (iMMR) in 2024, of 136.2 per 100,000 live births compared to the national average iMMR of 111.7 per 100,000 (13).

These mortality patterns highlight the important role that CHCs serve as the first point of contact for obstetric emergencies. Yet, CHCs have limited capacity to manage life-threatening complications, making timely upward referral essential (5). In practice, however, systemic inefficiencies frequently undermine this process, with many women arriving at higher-level facilities in more critical conditions (12). This paradox illustrates referral pathways existing on paper yet often functioning poorly in reality.

While existing literature shows the importance of effective referral systems (14–16), there is little evidence on the action and interaction strategies that frontline health workers employ to navigate these constraints in resource-limited contexts. Understanding these strategies is central for strengthening the effectiveness of upward referral of obstetric emergencies and reducing preventable maternal and neonatal deaths in the Eastern Cape.

## 3 Purpose of the article

Based on data from a broader grounded theory study, this article analyses the action/interaction strategies employed by frontline health workers to facilitate effective upward referral of obstetric emergencies from CHCs.

## 4 Methodology

### 4.1 Research design, approach and paradigm

Underpinned by a social constructivist (interpretivist) paradigm (35), this study adopted a qualitative approach and a GT design, guided by the Straussian/Corbin methodology, to explore action/interaction processes used by frontline health workers to facilitate effective upward referral of obstetric emergencies. Grounded Theory is particularly suitable for generating new insights into complex phenomena that are poorly understood (11, 36). In this study, GT provides a systematic approach to uncover the action and interaction strategies employed by frontline health workers.

### 4.2 Setting description

Research was carried out in the Eastern Cape province, in the Oliver Reginald (OR) Tambo district. The district has one of the significantly highest maternal mortality rates in South Africa, at 190 per 100,000 live births (13). Within this district, the municipality selected for the study faces an even higher maternal mortality ratio of 378.5 per 100,000 live births (37), which are the most recent publicly available municipality-level data.

The province is characterised by geographic and socio-economic challenges. It has a dispersed rural settlement pattern, with a population exceeding 7.2 million and population density of about 41.3 persons/km^2^ (17). It faces persistent challenges in infrastructure and service delivery, including limited access to water, sanitation, electricity and transport (17), all which influence health care access and referral pathway functioning. Although these features are largely contextual, they shape the operational realities for health services, especially in rural and resource constrained communities.

### 4.3 Population and sampling

The study employed both purposive and theoretical techniques, consistent with GT, to ensure that the chosen participants could offer rich and significant data (38). Midwives, medical officers (MOs) and Emergency Medical Services (EMS) paramedics were selected owing to their frontline and crucial responsibilities in managing and referring obstetric emergencies. Initial purposive sampling was followed by theoretical sampling which the initial participants guided. Theoretical sampling examining for new concepts, codes or growing existing codes continued until redundancy was reached (39). The sample comprised of thirteen (13) focus group discussions (FGDs) each with three to five midwives in accordance with Foley and Timonen (18) GT recommendations. In addition, eight (8) in-depth interviews (IDIs) were held with medical officers and three (3) with paramedics, totalling twenty-four (24) data collection sessions.

### 4.4 Data collection methods and process

Data were collected from August 2024 to May 2025 through document analysis, observations, FGDs and IDIs, using respective data collection guides. The purpose of the extensive data collection was to ensure methodological rigour and facilitate the constant comparison of findings, essential for the development of an emergent theory (38). Document analysis was done simultaneously to the observations. Both FGDs and IDIs were conducted in both English and isiXhosa, with FGDs lasting 35 min to an hour and IDIs 35 to 45 min. All FGDs and IDIs were verbatim transcribed by the ZJ (the principal researcher), who double-checked their accuracy with the supervisor (OBB) and research assistant (EW). To ensure coding uniformity, ZJ, OBB and EW frequently discussed their initial individual judgments together. Data collection totalled 24 sessions, until theoretical saturation was reached, evidenced by the absence of new categories and a focus on conceptual completeness, rather than predetermined sample numbers (39).

### 4.5 Data analysis

Data analysis followed Grounded Theory Strauss and Corbin principles with coding occurring in phases: open, axial and selective coding and constant comparative analysis throughout (38). Data collection and analysis were intertwined, allowing the researcher to iteratively refine concepts as new insights emerged from FGDs, IDIs, observations and document analysis. Analysis was led by ZJ and conducted manually. OBB and EW reviewed coding decisions and participated in discussions to ensure consistency in interpretation.

Open coding involved ZJ conducting line by line reading of the interview transcripts and field notes to identify initial codes, which were compared across data sources to find similarities, differences and patterns. OBB and EW independently reviewed to confirm alignment. Codes were constantly compared to confirm that they were grounded in the data rather than forcing already constructed codes upon the data. Axial coding followed, allowing ZJ to refine and organise codes into categories and subcategories that captured interaction strategies used by frontline health workers. OBB and EW reviewed the developing categories and debated areas of divergence with ZJ. During selective coding, ZJ refined and integrated the categories and subcategories identified during open and axial coding. This phase focused on understanding the relationships and interactions between different action and interaction strategies used by frontline health workers in upward referral. Where differences arose, consensus was reached through deliberation and agreement, ensuring that coding decisions and emerging categories were both rigorous and trustworthy.

Constant comparison was applied to ensure that the emerging patterns were coherent and grounded in the data, while theoretical sensitivity was maintained to remain open to new insights. This approach allowed a detailed analysis of strategies shaping referral processes, forming the foundation for future model development. Emerging concepts and categories were compared with relevant literature to enhance theoretical sensitivity and situate the findings, without directing the coding process.

### 4.6 Rigour

Trustworthiness was ensured through Lincoln and Guba (40)'s strategies of credibility, dependability, confirmability, transferability and triangulation. Credibility was established through prolonged engagements, by member checking, detailed field notes and engagements data triangulation. Dependability was achieved through maintaining an audit trail of a detailed description of the research methodology and data analysis. Confirmability was ensured through authenticating transcriptions and audio recording, field notes documentation, collecting data until saturation, independent data coding of data by ZJ and OBB, with agreement reached through discussions on emerging themes and by providing a thorough explanation of the researcher's design and methodology to reduce bias. For transferability, ZJ provided a detailed description of the research objectives, methods, settings and findings. Triangulation was employed by combining diverse data collection methods and sources, allowing for cross-verification.

### 4.7 Ethical considerations

Ethical clearance was obtained from Biomedical Ethical Research Committee (BREC) at the University of Kwa-Zulu Natal (reference number: XXX). Approval was obtained from the relevant authorities (departmental heads) and the participants themselves. Declaration of consent was provided, as well as detailed informational letters outlining the details of the study. Ethical considerations were comprehensively discussed in these communications. Participants were informed that participation was entirely voluntary and that they could withdraw at any point without facing any consequences. The South African Depression and Anxiety Group's (SADAG) contact details were provided to keep participants from going through emotional or psychological suffering because of the delicacy of obstetric crises. There were no documented cases of discomfort necessitating referral, demonstrating the precautions taken to ensure the participants' safety. All data is handled with strict confidentiality protocols and stored on a password-protected computer that only the researcher and authorised supervisors can access. Five years following the conclusion of the study, any residual electronic files will be permanently deleted in compliance with data protection procedures.

## 5 Findings

Table 1 lists all study participants under pseudonyms to maintain confidentiality. However, not all participants are quoted in the findings, as only the excerpts that best reflect the key themes were included.

Table 2 presents the categories, subcategories, illustrative quotes and document extracts that reflect the action/interaction strategies described by the participants.

**Table 2:** Action/interaction strategies that shape upward referral of obstetric emergencies from CHCs in eastern cape.

Category	Subcategory	Quotes	Document Extract
Improvised transportation	Community & alternative transport solutions	“*We sometimes ask families to hire their own private cars before the condition worsens because there are no ambulances*” (Khanyisile, MO, IDI 7)	In reviewing the referral registers, I observed that alternative transport methods were used in cases where ambulances were unavailable. For example, some entries noted “Private car used due to no ambulance” while in other cases, the mode of transport was not recorded. These observations suggest possible delays in emergency referrals and highlight gaps in transport documentation and coordination.
		“*Some patients come from deep rural areas. They don’t have money or families, so we pool funds for their transport because we have referred, but there is no ambulance*” (Jamjam, Hospital 1 midwife, FGD 11)	
		*“Sometimes when the ambulance can’t reach the CHC because the bridge is flooded, we get help from community members. They use a wheelbarrow to bring the woman across so that we can continue with the referral to hospital”* (Mandy, paramedic, IDI 9)	
	Emergency logistical adaptations	*“Sometimes we have had to send someone by foot to the admin block just use the landline because we are dealing with high-risk cases and the delay is life changing”* (Nomsa, Hospital 1, FGD 11)	Referral registers documented instances of delayed calls due to limited communication infrastructure, highlighting challenges in coordinating timely emergency transport. For example, some entries noted “Unable to reach ambulance on landline; used personal phone” or “Delay in referral due to no network coverage,” illustrating the communication barriers faced by staff
		*“The infrastructure is poor and there is an emergency, so you have put the patient on your and another person’s shoulder then walk to the response vehicle”* (Ntombifuthi, paramedic, IDI 11)	
		*“We recently had antepartum haemorrhage and there were road closures en-route to XXX hospital that we did not know of, we had to call police to escort us”* (Kwanda, paramedic, IDI 10)	
Informal communication networks	Personal phones & airtime sharing	*“It’s not easy at all. Sometimes our telephones are not working. You must use your own cell phone and airtime to facilitate the referral”* (Zoe, CHC 4 midwife, FGD 4)	In reviewing the medical case records (MCRs), I observed that staff relied on personal cell phones and WhatsApp for communication due to the unavailability of facility phones, with some with some highlighting “No landline available, used own phone”
		*“…make a plan and ask people for airtime because you have to call EMS and the hospital”* (Frank, CHC 3 midwife, FGD 3)	
		*“Even when we use our own cell phones, we stand at certain areas where there is connectivity to ensure we get a hold of EMS”* (Weliswa, CHC 2 midwife, FGD 2)	
	Remote coordination	*“…we also advise them remotely and tell the midwife to give magnesium sulphate, keep the woman lying on her left side, etc. when ambulances are delayed”* (Langa, MO, IDI 6)	In reviewing the referral registers, I observed that staff often used WhatsApp for remote case advice. For example, some entries noted “Consulted senior doctor via WhatsApp,” while in other instances, the mode of communication was not recorded. This highlights both the reliance on digital communication tools and gaps in documentation of remote coordination
		*“We have our own WhatsApp group. When you have trouble on the road, you can call, tell them what you are looking at now and ask for advice on what to do”* (Kwanda, paramedic, IDI 10)	
		*“We try to note everything on WhatsApp and try to have some of the people from the receiving and referral facilities are in that group and make calls enquiring about beds and ambulance availability”* (Mandy, paramedic, IDI 9)	
Unofficial documentation practices	Personal notebooks & logbooks	*“As you can see here, to assist ourselves we have designed this book in a way that shows what time the first call was made, second call, etc so we can follow up on referrals properly”* (Mandisa, CHC 5 midwife, FGD 5)	In reviewing medical case records (MCRs) and midwives’ personal notebooks, I observed that MCR entries occasionally lacked complete information on patient interventions prior to transfer. For example, some handwritten notes in personal logbooks provided details on vital signs, timing of interventions, and follow-ups of calls for ambulance transport. These observations highlight the complementary role of personal records in documenting patient care when official records are incomplete.
		*“We have this page where we note down the doctors’ numbers whenever we get them so we can get hold of them immediately when facilitating the referral because we do not get updated when they change them”* (Kwanele, MO, IDI 8)	
		*“I keep a personal notebook noting patient vital signs and medications given when official referral forms are missing so that the receiving hospitals knows exactly what has been done”* (Zoleka, Hospital 1 midwife, FGD 6)	
	Visual/digital evidence sharing	*“When we have photos of her CTG or IV setup sent from the CHC, this allows us to quickly review what’s been done, anticipated complications and continue care without delay”* (Sesethu, Hospital 3 midwife, FGD 19)	In reviewing medical case records (MCRs), I observed that visual documentation, such as photos, was sometimes sent to the receiving hospital for pre-transfer assessment. For example, some entries noted “Photo of CTG sent to referral hospital before transfer,” illustrating how staff used digital tools to support clinical decision-making and enhance pre-transfer communication
		*“We sometimes take pictures of CTG tracings, or the IV line setup on phones and send to the hospital, so the receiving team can see what’s covered before transfering*” (Nomzamo, Hospital 2 midwife, FGD 12)	
		*“Before the shift ends, we put up color-coded notes on the board to show which mothers are waiting for referral, who is urgent and what care has already been given. It helps the next team pick up the referral process without delays”* (Nosiphiwo, Hospital 2 midwife, FGD 8)	
Bedside improvisation	Dynamic case prioritisation	*“We sometimes have to prioritise which patients get transferred first, so the most critical cases, like severe haemorrhage, are referred immediately while others wait”* (Lunga, Hospital 1 midwife, FGD 6)	In reviewing the referral registers and medical case records (MCRs), I observed that staff often prioritised cases based on urgency. For example, some entries noted that “Critical patient attended first” or “Stable patient advised to wait for ambulance,” indicating that decisions about patient transfer and care were dynamically adjusted according to the severity of the condition. These observations demonstrate how staff triaged cases in real time to manage limited resources and ensure timely care for the most urgent patients
		*“When a critically ill woman arrives, like one with severe haemorrhage, we immediately stabilise her and prepare the team and resources, so the referral is completed safely and urgently, even if other ill patients are waiting*” (Nkosazana, Hospital 3 midwife, FGD 10)	
		*“Sometimes a referred patient arrives needing urgent care, like a blood transfusion, but essential equipment is limited. We then quickly coordinate across wards and improvise with what we have to ensure the referral is completed safely and without delay”* (Abonga, Hospital 2 midwife, FGD 7)	
	Adaptive clinical practices	“*When suction equipment is broken, we use manual techniques to clear the airway and keep the patient stable until transfer”* (Zikhona, CHC 1 midwife, FGD 1)	In reviewing the medical case records (MCRs), I observed that staff adapted clinical practices to meet patient needs despite resource limitations. For example, some entries noted that “IV fluids administered using available supplies” or illustrating how staff employed flexible strategies to maintain patient care. These observations highlight the resourcefulness and adaptability of clinical staff in challenging circumstances.
		*“Even if there isn’t a formal booking, we sometimes contact the blood bank or theatre to hold resources before the patient arrives, so that urgent referrals can be managed immediately”* (Zakhele, MO, IDI 4)	
		*“Because of severe transport delays and long travel times, we sometimes have to manage two patients at once, stabilising them so both can continue their referral safely to the hospital”* (Kwanda, paramedic, IDI 10)	
Resource reallocation	Human resource adaptation	*“We currently have a doctor here for two weeks, for training, we are using her as an extra pair of hands to assist with patient care so that women needing referral assessments aren’t delayed”* (Fefekazi, MO, IDI 3)	In reviewing medical case records (MCRs) and referral registers, I observed that staff adapted human resources to meet patient care needs. For example, some entries noted “Midwife on duty covered additional shift” or “Staff reallocated to support emergency referral,” illustrating how personnel responsibilities were adjusted to ensure continuity of care. These observations highlight the flexible allocation of human resources in response to service demands.
		*“When staff learn new skills, such as performing dating scans, they can manage more cases at the CHC, reducing unnecessary referrals to the hospital and speeding up care for those who truly need it”* (Sandiso, MO, IDI 2)	
		*“Sometimes, when staff are limited, we take on multiple roles and work double shifts to ensure referred patients receive timely care and their transfer to the hospital isn’t delayed”* (Wazini, Hospital 1 midwife, FGD 11)	
	Resource sharing & redistribution	*“When a referred patient needs monitoring, but we don’t have enough CTGs, we borrow machines from nearby facilities to ensure the referral is completed safely*” (Natasha, Hospital 1 midwife, FGD 6)	In reviewing staff reports and handover notes, I observed that equipment and beds were occasionally rearranged to support timely patient care. For example, some entries reflected temporary repositioning of resources to accommodate emergencies or improve patient flow. These observations suggest an adaptive use of physical resources, although such adjustments were not consistently or formally documented.
		*“When a referred mother arrives or needs transfer and the ambulance lacks essential equipment like oxygen or a monitor, we quickly borrow what’s needed to ensure the patient can be safely transported to the hospital”* (Nqobile, Hospital 2 midwife, FGD 8)	
		*“Multiple high-risk patients are sometimes referred to us, so we sometimes redistribute beds and move patients around so that those who need urgent care or monitoring can be accommodated immediately, to support timely referral management”* (Nokwanda, Hospital 3 midwife, FGD 13)	
Adapting referral protocols	Streamlining paperwork	*“Sometimes we refer without waiting for an official letter because the case is urgent.”* (Lelethu, CHC 5 midwife, FGD 5)	Midwives’ notes showed several cases where patients were received without formal referral letters. Instead, the notes reflected only that the patient had arrived and care was initiated, with no accompanying documentation from the referring facility. This points to inconsistent adherence to referral documentation requirements.
		“*We bypass levels and call a doctor directly at the hospital to speed things up.”* (Nozibele, CHC 2 midwife, FGD 2)	
		*“It is so common to accept patients without referral letters to avoid life-threatening delays”* (Busisiwe, MO, IDI 5)	
	Rule-bending for urgent referrals	*“We have to persuade ambulance staff or dispatch, often by calling someone we know at EMS, to prioritise a critically ill patient over routine transfers, ensuring urgent cases reach the hospital in time”* (Florence, CHC 4 midwife, FGD 4)	While EMS records indicated that certain patients were rerouted to better-resourced facilities in response to case severity, the basis for prioritising these cases was not consistently documented. This lack of justification creates gaps in the referral trail.
		“*We sometimes have to bypass a closer but under-resourced facility for a hospital better equipped to manage the complications”* (Mandy, paramedic, IDI 9)	
		*“I often instruct interns to meet ambulances at casualty instead of waiting for paperwork, especially when a mother arrives with severe postpartum haemorrhage so she can receive care immediately”* (Sandiso, MO, IDI 2)	

### 5.1 Improvised transportation

#### 5.1.1 Community and alternative transport solutions

Midwives described how they strategically mobilised families to hire private vehicles when ambulances were unavailable and pooled funds to secure alternative transport to prevent further deterioration of the patient's condition. Referral registers also indicated instances where alternative transport was used due to ambulance unavailability, although in some cases the transport method was not recorded. Additionally, paramedics reported on collaboration with local communities to overcome physical barriers to access, such as relying on wheelbarrows to move women across flooded areas so that the referral process continues.

#### 5.1.2 Emergency logistical adaptations

Participants indicated that in high-risk cases they sometimes dispatched staff on foot to access a landline in the administration block. Paramedics described situations where poor infrastructure required them to improvise by carrying women on their shoulders to reach emergency vehicles. Additionally, they highlighted how road closures necessitated police escorting ambulances. Document analysis corroborated these accounts with referral registers presenting instances of delayed calls due to limited communication infrastructure.

### 5.2 Informal communication networks

#### 5.2.1 Personal phones and airtime sharing

When formal communication systems failed, midwives reported using personal phones and airtime to contact ambulances and receiving facilities. They also strategically positioned themselves in areas with stronger reception to maximise the chances of making contact for referral. This practice was also observed during fieldwork, where midwives relied on their personal phones because some CHC had no functioning landlines at all. The absence of institutional communication tools compelled them to improvise.

#### 5.2.2 Remote coordination

MOs explained that when ambulances were delayed, they guided midwives in stabilising patients, for example instructing them to administer magnesium sulphate. Paramedics mentioned the use of WhatsApp groups to share real-time challenges encountered en-route and receiving immediate clinical advice. Referral registers often reflected such communication for remote case guidance, although in many instances the specific mode of communication was not recorded.

### 5.3 Unofficial documentation practices

#### 5.3.1 Personal notebooks and logbooks

Participants stated that they designed personal logbooks to track the timing of referral calls to systematically follow-up when delays occur. MOs highlighted how they compiled contact lists of doctors in personal notebooks, countering the lack of updated official directories and ensuring immediate access during emergencies. Such ingenuity was visible during fieldwork, where midwives displayed self-designed logbooks used to document call times and outcome. Others described keeping detailed patient notes to hand over when official referral forms were unavailable. Document analysis showed that MCR entries occasionally lacked complete information on patient interventions prior to transfer, so supplementary handwritten notes maintained by CHC staff sometimes provided details on vital signs and timing of ambulance calls and follow ups, which were referenced during handover at the receiving facility.

#### 5.3.2 Visual/digital evidence sharing

Participants reported sending photos of CTG tracings and IV setups to receiving hospitals to prepare in advance. MCR reviews confirmed this practice, noting that photos were routinely sent to hospitals for pre-transfer assessment. Within facilities, staff used colour-coded notes on ward boards to indicate referral status, level of urgency and care already provided, allowing incoming teams to immediately orient themselves and continue the referral process without interruption.

### 5.4 Bedside improvisation

#### 5.4.1 Dynamic case prioritisation

Midwives explained that when women presented with life-threatening conditions such as severe haemorrhage, they stabilised patients and mobilised teams and resources immediately, even by delaying less urgent cases. Due to resource scarcity, they coordinate across wards and repurpose available equipment to make urgent transfers possible.

#### 5.4.2 Adaptive clinical practices

When suction devices failed participants improvised manual airway clearance techniques, ensuring safe stabilisation. MOs described pre-emptively liaising with blood banks and theatres to reserve critical resources before critical patients arrived to be managed without delay. Paramedics recounted situations where severe transport delays required them to stabilise and monitor multiple patients simultaneously.

### 5.5 Resource reallocation

#### 5.5.1 Human resource adaptation

When additional personnel such as trainee doctors, were temporarily available, they were strategically integrated into patient care to prevent referral assessment delays. Additionally, newly acquired skills, such as performing dating scans, were applied to manage more cases locally reducing unnecessary referrals. During fieldwork, a doctor who was initially present to learn to operate newly introduced equipment was also drawn into referral-related tasks. In times of acute shortage, participants reported taking on multiple roles and extended shifts, ensuring that women requiring urgent transfers were not left unattended.

#### 5.5.2 Resource sharing and redistribution

Midwives explained that when essential equipment such as CTG machines, oxygen, or monitors was unavailable, they negotiated borrowing from nearby facilities and ambulances, so urgent transfers could proceed. Participants also recounted how they reorganised ward spaces and redistributed beds, prioritising high-risk women so that referral management and monitoring were not delayed. Staff handover notes occasionally indicated that equipment was temporarily rearranged to support timely care.

### 5.6 Adapting referral protocols

#### 5.6.1 Streamlining paperwork

Participants reported on sending patients without referral letters, phoning hospital MOs directly and receiving referrals without documentation as deliberate strategies to override bureaucratic delays, prioritising patient survival over procedural compliance. Observations confirmed that quick, direct phone communication helped prevent delays in emergency interventions. On the other hand, midwives' notes documented acceptance of patients without formal referral letters.

#### 5.6.2 Rule-bending for urgent referrals

Midwives sometimes persuaded and leveraged personal networks within EMS to prioritise critical transfers, paramedics bypass under-resourced facilities in favour of better-equipped hospitals, and MOs instruct interns to meet ambulances directly to avoid paperwork delays to accelerate access to emergency care. EMS records reflected instances where patients were diverted to better-equipped facilities due to case severity, although in some cases the rationale for prioritisation was not documented.

## 6 Discussion

The findings of this study revealed that healthcare providers in OR Tambo employ a variety of action/interaction strategies to overcome barriers in the obstetric emergency referral process. These strategies reflect the dynamic, adaptive and collaborative responses that align with Strauss and Corbin's (11) GT framework, particularly the micro-level interactional processes enacted under contextual constraints. The categories demonstrate how frontline health workers engage in purposeful action and problem-solving within the context of limited resources, reflecting both individual and collective agency in sustaining the referral system.

Participants described improvised transportation strategies to maintain upward referral when formal systems were unavailable. Midwives mobilised families to hire private vehicles, pooled financial resources to secure alternative transport and collaborated with local communities to overcome physical barriers, such as using wheelbarrows to traverse flooded areas. In high-risk cases, staff sometimes walked to administration blocks to access landlines, carried patients on their shoulders to reach ambulances, or coordinated with police to navigate road closures. These actions illustrate adaptive strategies that arise in response to systemic constraints. Where ambulance services are limited, frontline staff and community members actively problem-solve to ensure patients reach higher-level care. Similar patterns are observed in other low-resource settings. In Ghana and Uganda, families and healthcare workers often rely on taxis, motorbikes, or other improvised transport when formal ambulance services are unavailable (3, 19, 20). Globally, Sacks et al. (21) note that obstetric and newborn transport frequently remains the responsibility of families, reinforcing that informal, collaborative transportation strategies are critical for maintaining continuity in emergency obstetric referrals. In South Africa, the observed absence of transport modality documentation in referral registers has implications beyond individual cases. Incomplete recording of transport modalities limits the Department of Health's ability to accurately audit referral delays, quantify unmet ambulance demand (22) and generate evidence-based justifications for additional transport resources, potentially perpetuating systemic under-resourcing. Building from these immediate, practical improvisations in transportation, frontline workers also engaged in communication-based interactional strategies to maintain coordination and continuity of care.

When formal communication systems were unreliable, due to faulty landlines or limited office resources, participants enacted informal communication networks as an adaptive strategy to maintain referral coordination. They used personal mobile phones, airtime and WhatsApp groups to contact referral hospitals, pre-alert teams and obtain guidance from doctors, enabling faster decision-making and continuity of care. Comparable practices have been documented in other low-resource contexts such as Ghana where WhatsApp has strengthened communication between primary facilities and hospitals, improving feedback and referral tracking (16, 20). In Uganda, personal phone calls are routinely used to facilitate pre-referral discussions and obtain guidance when doctors are absent (23) as well as in Northern Iraq (24). By improvising informal networks, participants were able to circumvent systemic gaps and ensure the continuity and responsiveness of emergency referrals. Beyond communication, participants' strategies evolved to encompass more structured improvisations through informal documentation, which sustained accountability and continuity amid system inefficiencies.

Participants reported developing informal documentation systems, including personal logbooks, contact lists and visual aids such as colour-coded ward boards, to track referral progress and patient status. The absence of formal tools triggered adaptive action, resulting in practical, locally generated solutions that bridged institutional gaps. Evidence from Ghana reflects similar patterns where incomplete or missing referral forms forced health workers to rely on follow-up calls or improvised notes (25), while in Uganda, healthcare workers maintained personal notebooks to track referral details and support continuity of care (23). Comparable trends have also been noted in other South African district hospitals, where staff developed informal tracking systems to compensate for inconsistent referral communication (16), highlighting that this is not an isolated occurrence. Together, these findings highlight strategies of frontline staff actively generating informal documentation mechanisms to preserve critical clinical information and facilitate timely, coordinated referral processes. These documentation innovations were accompanied by bedside improvisations that demonstrated rapid, situational decision-making to ensure timely and safe patient transfers.

Faced with life-threatening complications, such as severe haemorrhage, midwives stabilised patients by mobilising teams and repurposing equipment immediately, even if less urgent cases were delayed. Participants coordinated across wards, pre-emptively liaised with blood banks and theatres and improvised clinical procedures, such as manual airway clearance when suction devices failed, to ensure timely access to critical resources. Paramedics reported stabilising and monitoring multiple patients simultaneously during prolonged transport delays. Comparable patterns have been observed in other contexts. In Uganda, healthcare workers similarly coordinate across wards and pre-emptively prepare resources to manage high-risk referrals (23). Interestingly, New Zealand documented similar improvisation where EMS teams adaptively respond to equipment limitations and long transport distances (26), illustrating that such strategies are employed globally. The practices reflect a process of actions and interactions in response to the condition of resource scarcity and high patient acuity where frontline staff dynamically assess risk, allocate tools and employ improvisation to effective referrals. Moving from bedside-level improvisations, the data show how staff restructured human and material resources to sustain system functioning under persistent constraints. While bedside improvisation was frequently lifesaving, it also carries potential clinical risks, including compromised infection prevention practices (27), deviations from standardised protocols (28) and medico-legal vulnerability for providers (29). The findings therefore point not to the elimination of improvisation, but to the need for health systems to support 'safe improvisation'.

Participants described strategic reallocation of human and material resources to maintain timely referral and patient care under constrained conditions. Temporarily available personnel, such as trainee doctors, were integrated to support patient assessment, while staff applied newly acquired skills, like dating scans, to manage cases locally and reduce unnecessary transfers. During acute shortages, healthcare workers took on multiple roles and extended shifts, ensuring continuity of care. Material resources were also redistributed: essential equipment CTG machines, oxygen and monitors was borrowed from nearby facilities or ambulances and ward spaces and beds were reorganised to prioritise high-risk patients. In Uganda, staff similarly leveraged in-service training and skill-sharing to improve pre-referral management and reduce unnecessary transfers (23). In Nigeria, personnel and equipment were reassigned across wards to manage multiple high-risk obstetric cases simultaneously (30). Even in high-resource contexts, such as New Zealand, EMS personnel actively deploy staff and mobile resources to respond to multiple pre-hospital emergencies (26). Finally, the discussion culminates with action/interaction strategies involving adaptive modification of referral protocols where frontline staff challenge procedural rigidity to prioritise patient survival.

These adaptive strategies, while essential, may also contribute to provider fatigue, moral distress, and burnout, particularly where staff routinely use personal resources such as airtime, transport funds, and extended shifts (31). Moreover, as aforementioned, bypassing formal referral protocols exposes providers to medico-legal risk and introduce variability in care. The recognition of such points to institutionalising support mechanisms rather than relying on sustained individual sacrifice.

Data sources described deliberate adaptations to standard referral procedures to overcome bureaucratic delays and prioritise patient survival. Strategies included sending patients without referral letters, directly contacting hospital medical officers and leveraging personal networks within EMS to accelerate transfers. Midwives persuaded colleagues and used informal channels to prioritise critical cases, while paramedics sometimes bypassed under-resourced facilities in favour of better-equipped hospitals. Medical officers instructed interns to meet ambulances directly, circumventing procedural bottlenecks and ensuring timely access to emergency care. These practices show how frontline staff balance following referral rules with keeping patients safe, adjusting the rules as needed to fit resource constrained settings. Similar strategies have been observed in Ghana and Mozambique, where healthcare providers circumvented formal procedures to facilitate urgent referrals and prevent delays in life-threatening cases (20, 32, 33). In essence, professional judgment and collaborative networks enabled healthcare workers to maintain continuity and quality of care in resource-limited environments.

### 6.1. Limitation

This study focused on the perspectives of midwives, medical officers and paramedics working in public hospitals within the OR Tambo District of the Eastern Cape. As with grounded theory research, the findings are not intended for statistical generalisation to other provinces or countries with different referral systems, resources and health system structures. However, the contextual conditions described, rural dispersion, ambulance shortages, staffing constraints and infrastructural limitations are shared across other rural districts in the Eastern Cape and provinces such as Limpopo, as well as in comparable low- and middle-income settings. From a grounded theory perspective, the emergent concepts and action/interaction strategies developed here provide theoretical insights that may be transferred and adapted to other resource-constrained health systems with similar contextual challenges. Thus, although contextually bounded, the study's theoretical contribution strengthens understanding of how frontline health workers navigate systemic constraints in emergency obstetric referral processes.

### 6.2. Recommendations

- Recognising WhatsApp and similar secure messaging tools as official communication channels can enhance real-time coordination between CHCs, hospitals and ambulance services. Any formalisation should be aligned with South Africa's Protection of Personal Information Act (POPIA), including clear guidance on informed consent, de-identification of patient information and images, secure device use and institutional accountability for data governance.
- To manage high-demand periods and prevent delays in urgent referrals, systems should allow temporary deployment of additional doctors, paramedics, or trained personnel to facilities experiencing acute service pressure.

## 7. Conclusion

This article analysed the action and interaction strategies employed by MOs, midwives, and EMS paramedics in facilitating the upward referral of obstetric emergencies from CHCs. The findings, grounded in GT, revealed that despite constraints, healthcare workers consistently went beyond formal protocols to ensure timely and effective emergency care. The efforts were reflected in improvised transportation, informal communication networks, the use of unofficial documentation practices, bedside improvisation, reallocating resources and adapting referral protocols. The study highlights the critical role of flexibility, innovation and interprofessional collaboration in overcoming systemic barriers to emergency referrals. Future interventions should formalise effective informal strategies, such as WhatsApp communication and strengthen staffing measures during high-demand periods to enhance referral efficiency and maternal outcomes.

## Data Availability

The datasets generated and analysed during this study are not publicly available due to ethical restrictions protecting participant confidentiality. Full transcripts could risk indirect identification of participants and facilities. The study is ongoing, with additional publications planned; unrestricted public release at this stage could compromise future analyses. The Biomedical Research Ethics Committee of the University of KwaZulu-Natal approved the study, including procedures for secure data handling. De-identified excerpts may be made available upon reasonable request for academic research purposes, subject to approval by the University of KwaZulu-Natal and compliance with ethical requirements. Requests should include a brief description of intended use and be directed to the corresponding author at finaljuqu@gmail.com.
